# A Combined Morphological and Molecular Evolutionary Analysis of Karst-Environment Adaptation for the Genus *Urophysa* (Ranunculaceae)

**DOI:** 10.3389/fpls.2021.667988

**Published:** 2021-06-10

**Authors:** Deng-Feng Xie, Rui-Yu Cheng, Xiao Fu, Xiang-Yi Zhang, Megan Price, Yan-Ling Lan, Chang-Bao Wang, Xing-Jin He

**Affiliations:** ^1^Key Laboratory of Bio-Resources and Eco-Environment of Ministry of Education, College of Life Sciences, Sichuan University, Chengdu, China; ^2^College of Science, Jiamusi University, Jiamusi, China

**Keywords:** adaptive evolution, positive selection, transcriptome, *Urophysa*, karst environment

## Abstract

The karst environment is characterized by low soil water content, periodic water deficiency, and poor nutrient availability, which provides an ideal natural laboratory for studying the adaptive evolution of its inhabitants. However, how species adapt to such a special karst environment remains poorly understood. Here, transcriptome sequences of two *Urophysa* species (*Urophysa rockii* and *Urophysa henryi*), which are Chinese endemics with karst-specific distribution, and allied species in *Semiaquilegia* and *Aquilegia* (living in non-karst habitat) were collected. Single-copy genes (SCGs) were extracted to perform the phylogenetic analysis using concatenation and coalescent methods. Positively selected genes (PSGs) and clusters of paralogous genes (Mul_genes) were detected and subsequently used to conduct gene function annotation. We filtered 2,271 SCGs and the coalescent analysis revealed that 1,930 SCGs shared the same tree topology, which was consistent with the topology detected from the concatenated tree. Total of 335 PSGs and 243 Mul_genes were detected, and many were enriched in stress and stimulus resistance, transmembrane transport, cellular ion homeostasis, calcium ion transport, calcium signaling regulation, and water retention. Both molecular and morphological evidences indicated that *Urophysa* species evolved complex strategies for adapting to hostile karst environments. Our findings will contribute to a new understanding of genetic and phenotypic adaptive mechanisms of karst adaptation in plants.

## Introduction

Environmental heterogeneity is one of the most important factors influencing evolutionary trajectories and ecological adaption of species (Allouche et al., 2012). Plant species have adapted to pronounced gradients of environmental conditions (temperature, drought, oxidative, or osmosis stresses etc.) along with variability in their natural habitats. Species survival is dependent on maximizing their fitness in all environmental conditions they encounter (Pratlong et al., 2015). Therefore, understanding the drivers and genetic mechanisms of species persistence and adaptation is a basis for the study of ecological adaption, evolution, and speciation (Schnitzler et al., 2011).

The area of karst in southern and southwestern China boasts over 20,000 plant species, and its flora is ranked as one of the most endemic-rich subtropical flora in the world (Kang et al., 2014). This region is not only one of the most famous models of karst landform in the world with an area of 6.2 × 10^5^ km^2^ (Yuan, 1994; Su et al., 2002; Huang Q. et al., 2008), but also has the most varied of extreme karst environments characterized by low soil water content, periodic water deficiency and poor nutrient availability, and may have exerted strong selective forces on plant evolution (Ai et al., 2015). Although the ecological environment in karst region is extremely hostile, plant communities in this region exhibit remarkably high levels of species richness and endemism and make a large contribution to the floristic diversity of China (Gao et al., 2015; Hao et al., 2015; Feng et al., 2020). Moreover, this region possesses various ecological niches afforded by complex terrains (e.g., fissured cliffs and extensive caves) and variable climatic and edaphic conditions (Hao et al., 2015). Soils in this area are also typically shallow and are characterized by higher concentrations of calcium (Ca^2+^) and magnesium (Mg^2+^), higher pH levels, and a lower water storage capacity compared to non-karst soils in other subtropical or tropical regions (Nie et al., 2011; Luo et al., 2012). Many calcicoles (species adapted to calcareous soil) have evolved in the region, and their habitats and niches have long been regarded as ‘natural laboratories’ for ecological and evolutionary studies to understand natural selection and species evolution because of the high diversity and unique biota (Clements et al., 2006). Unfortunately, previous studies on karst-specific species are scarce (Kang et al., 2014; Ai et al., 2015; Li et al., 2015; Liu et al., 2019; Feng et al., 2020), and until now, the mechanisms of karst environmental adaptation have rarely been examined. Yet such information is important for understanding the survival and adaptive strategies of species in the special karst environmental conditions.

**FIGURE 1:**
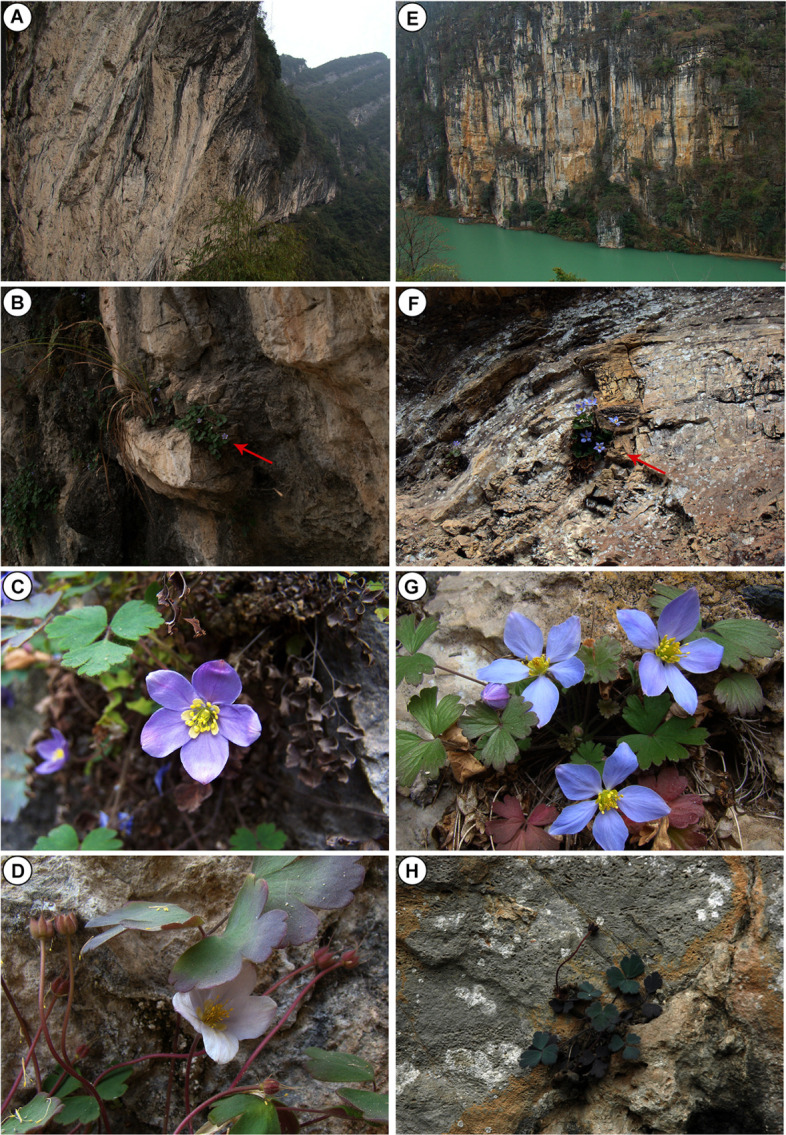
The habitat and morphological characters of *Urophysa rockii* **(A–D)** and *Urophysa henryi* **(E–H)**. **(A,E)** Habitat feature; **(B,F)** growth sites; **(C,G)** the flowers; **(D,H)** fruits.

One particularly interesting genus endemic to the karst region is the *Urophysa* Ulbr., which belongs to the family Ranunculaceae. The genus consists of two species, *Urophysa rockii* Ulbrich and *Urophysa henryi* (Oliver) Ulbrich, which are distributed in allopatric regions and are adapted to remarkably uniform habitats with specific calcareous soils developed from karst limestone bedrock in southwest China (Figure 1) (Wang et al., 2004; Xie et al., 2017). *U. rockii* and *U. henryi* exhibit distinct petal morphologies: the former has sacs near the base of petals while the latter has not (Xie et al., 2017). Among them, *U. rockii* (also be called the Panda Grass) was first discovered in 1925 but was not found until Dr. Chunyu Li rediscovered it in Jiangyou County of western Sichuan Province in 2005 (Du et al., 2010). There are only approximately 2,000 individuals in five populations currently surviving in upstream of the Fujiang River (Wang and He, 2011; Xie et al., 2017). Hence it was listed as an endangered species and included on the China Species Red List. Only a few populations of *U. henryi* have been found and most are separated by high mountains and deep valleys in karst regions of southern China (Xie et al., 2016, 2017). Interestingly, the two species all flower in the winter (flourishing florescence is between December to March), and in field observations and laboratory experiments, we found that *U. rockii* and *U. henryi* can not survive outside the karst limestone, which indicated that the karst limestone plays a significant role in their growth and development. However, there have been no studies investigating the biological adaptation mechanisms in the two species, but there have been studies of their genetic diversity and differentiation (Xie et al., 2016, 2017), growing environment and conservation strategies (Liu Y.Q. et al., 2009; Du et al., 2010), and biological and ecological characteristics (Wang and He, 2011; Zhang L. et al., 2013; Zhang et al., 2013a, b). More comprehensive studies are required because populations of the two species have been lost, degraded and fragmented by human development activities (e.g., scenic spot construction, hydroelectric stations) and excessive exploitation due to their medicinal value (contusion and bruise treatment). Therefore, before more populations are lost, it is crucial to explore the influence of the karst-specific habitat on the evolution and growth of these two species.

With the rapid development of sequencing technologies, the whole-genome and transcriptome sequencing have become increasingly popular analytical techniques that can investigate various adaptive processes, such as in response to highland, salt, drought, and temperature stress (Li et al., 2014; Zhang et al., 2016, 2019, 2020; Mock et al., 2017; Wang K. et al., 2019; Zeng et al., 2019; Birkeland et al., 2020; Xie et al., 2020). However, the vast majority have been performed on animals and limited genome/transcriptome-based research has been devoted to plants in the karst region. Previous studies on the genus *Primulina* which has been studied the most in karst limestone habitats, indicated that genome size is phylogenetically conserved but its variation among species is a combination of both neutral and adaptive evolution (Kang et al., 2014; Feng et al., 2020). Tao et al. (2016) suggested that the Ca^2+^-permeable channel TPC1 may be involved in the local adaptation of *Primulina* to karst Ca^2+^-rich environments, and the potential positive selection in phytochrome PHYE may play an important role in the adaption of *Primulina* to the heterogeneous environment of karst (Tao et al., 2015). Nevertheless, we know little about the genetic bases of plants adapted to the karst environment.

In this study, we performed RNA-seq to obtain most transcriptome sequences of the karst species *U. rockii* and *U. henryi* and non-karst species in genus *Semiaquilegia* and *Aquilegia*. Single-copy genes were extracted for phylogenetic analysis, and by positive selection analysis and function annotation, we aimed to investigate the potential mechanisms of how these two-species adapted to the karst environment. This study represents the first exploration of *Urophysa* transcriptomes using the high-throughput RNA-seq, and will help guide further molecular systematics, population genetic, and ecological adaption studies in *Urophysa* and other related species.

## Materials and Methods

### Morphological Character Investigation, Sample Collection, and Transcriptome Sequencing

To understand the morphological characters and living conditions of the karst and non-karst species, we performed *in situ* and *ex situ* field investigation in the Sichuan, Hubei and Shanxi Provinces of China. The morphological traits and habitats of each species were recorded, including the flowering time, flower and leaf traits and color variation, seeds dispersal and living environment condition. The young and mature leaves and flowers of *U. rockii* and *U. henryi* as well as *Semiaquilegia adoxoides* and *Aquilegia ecalcarata* were collected from living plants, and stored at −80°C until processed for total RNA isolation. Total RNA was extracted using Trizol (Life Technologies Corp.) according to the manufacturer’s protocols, and cleaned up using the RNeasy mini kit (Qiagen, Valencia, CA, United States). Poly (A) mRNA was purified using Oligo (dT) magnetic beads and interrupted into short fragments. The RNA sequencing libraries were constructed using the Illumina mRNA-Seq Prep Kit after assessing RNA quality. Finally, all cDNA libraries were sequenced on the Illumina HiSeq 2000 platform in paired-end form. A total of eight sequencing libraries were constructed in this study, including each flower and leaf libraries of the four species.

### *De novo* Assembly, Completeness Test, and Functional Annotation

Quality of clean data (clean reads) was assessed using the FastQC version 0.11.8^1^ by removing sequencing adaptors and reads with unknown nucleotides and low quality (quality scores < 20). All subsequent analyses were based on these filtered reads. Transcriptome *de novo* assembly was performed using Trinity software with default parameters (Grabherr et al., 2011). To evaluate the completeness of the assembly results, we applied BUSCO version 3.0.2 (Simão et al., 2015) to assess the quality of assembled transcriptome data based on the embryophyte gene database (embryophyte_odb 10.2020-09-10)^2^. Only contigs with lengths greater than 200 bp were kept for further analysis. Then, CD-HIT-EST version 4.6.8 (Li and Godzik, 2006) was used to remove redundant contigs with a threshold of 1, and TransDecoder version 0.36^3^ was employed to predict the open reading frame (ORF) with a minimum protein length of 100 bp.

Functional annotations of all assembled protein sequences were performed by searching against the following databases: Flowering plant in NCBI Non-redundant protein sequence database (NR, Taxonomy ID is 3398), Protein family (Pfam), Clusters of Orthologous Groups of proteins (COG), Swiss-Prot protein (Swiss-Prot), KEGG ortholog database (KEGG), and Gene Ontology (GO). Among them, sequences were searched against the flowering plant database, Pfam, COG, and Swiss-Prot using BLASTP version 2.2.31 with an *e*-value cut-off of 1 × e^–5^. GO and KEGG annotations were performed by Blast2GO software (Conesa et al., 2005) with an *e*-value cutoff of 10^–5^ and then plotted with functional classification using Web Gene Ontology Annotation Plot (WEGO) (Ye et al., 2006).

### Orthologous and Paralogous Genes Identification and Phylogenetic Analysis

**FIGURE 2:**
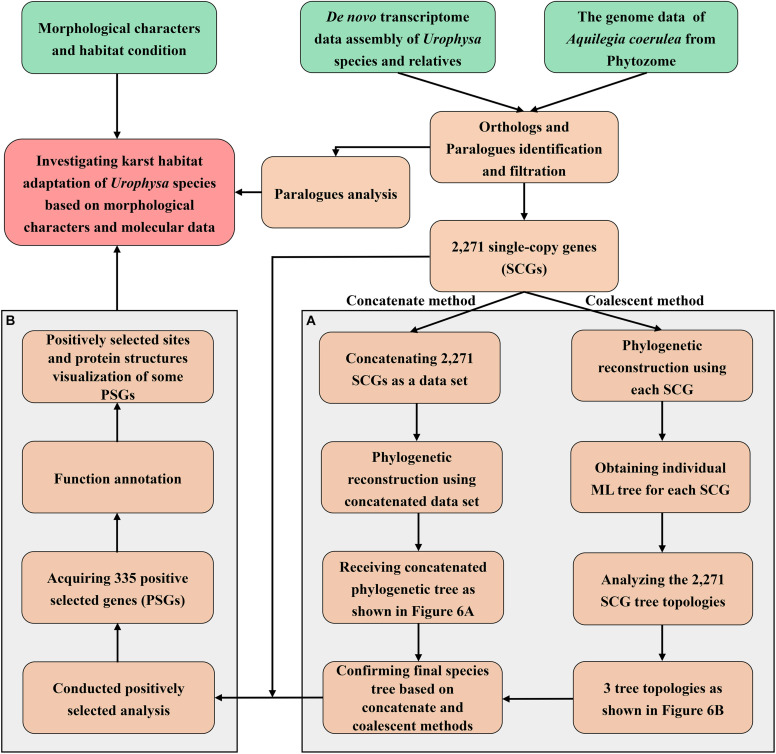
The detailed analysis procedures performed in this study. **(A)** The phylogenetic analysis workflow including concatenate and coalescent methods; **(B)** procedures of positive selection analysis.

In order to identify the shared orthologs and then used for further analyses, genome data of *Aquilegia coerulea* were downloaded from Phytozome^4^ and the OrthoMCL version 2.0.9 software (Li et al., 2003) was used to extract the orthologous genes of the five species with *e*-value of 1e^–10^ and inflation = 1.5. The one to one ortholog predictions were conducted according to the following criteria: (1) Muscle (Edgar, 2004) was used to align the sequences of each orthologous group from the flow of Agalma (Dunn et al., 2013); (2) GBlocks (Talavera and Castresana, 2007) was used to filter the conserved regions of each sequence; (3) the ML tree was built in IQ-TREE 2 (Minh et al., 2020) by using above filtered conserved sequences with parameters set as: iqtree -s ^∗^.phy -m MFP -bb 1,000 -bnni -cmax 20 -redo; (4) the ML tree was trimmed by implementing the Dendropy (Sukumaran and Holder, 2010) and only one copy of each species was finally obtained in each orthogroup. Additionally, PAL2NAL version 14 (Suyama et al., 2006) was employed to map the nucleotide sequences of each ortholog to the corresponding protein sequences and remove the highly variable nucleotide sites to obtain the final nucleotide sequences. A total of 2,271 single-copy genes (SCGs) were generated and concatenation and coalescent methods were implemented for phylogenetic analysis. Meanwhile, 243 clusters of paralogous genes were detected. For detailed analysis procedures, please see Figure 2.

For the concatenation method, all SCGs were concatenated as a super data matrix after alignment and manual checking. Two conventional approaches were used to perform the phylogenetic analysis: (1) A Maximum Likelihood (ML) tree was reconstructed using IQ-TREE 2 (Minh et al., 2020), with the parameters applied as: iqtree -s ^∗^.phy -m MFP -bb 1,000 -bnni -cmax 20 -redo. (2) A Bayesian inference (BI) analysis was performed in MrBayes v3.2 (Ronquist and Huelsenbeck, 2003) with the best-fit model GTR + G being selected under the Akaike information criterion (AIC). Four independent Markov chain Monte Carlo (MCMC) runs were conducted, each chain run for 5 × 10^8^ generations using initial trees with every 1,000 generations being sampled, and the initial 20% of the samples were discarded as burn-in to confirm the stationarity. Tracer v1.5 (Rambaut and Drummond, 2009) was used to assess the quality of the MCMC simulations and the stability of runs. Effective sample size (ESS) values were greater than 200 for all parameters, indicating sufficient sampling. For the coalescent method, we performed the ML analysis for each of 2,271 SCGs using IQ-TREE 2 (Minh et al., 2020) with the same parameters and processes as in the concatenation method. *Urophysa* species were rooted as outgroup based on previous studies (Xie et al., 2017; Zhai et al., 2019; Duan et al., 2020). Furthermore, in order to improve the average positive branch rates and achieve believable phylogenetic results, the programe STAR (Liu L. et al., 2009) and MP-EST (Liu et al., 2010) were used to estimate the species tree based on 2,271 gene trees. Finally, the concatenate and coalescent phylogenetic results were used for species tree derivation.

### Positive Selection Analysis

To detect positive selection on genes along a specific lineage, the optimized branch-site model (BSM) (Yang and Dos, 2011) in the Codeml program of the PAML 4 package (Yang, 2007) was performed. This model was conducted based on (1) a confirmed phylogenetic tree; (2) gene sequences with Paml format; (3) parameter-setting file; (4) Bayesian empirical Bayes (BEB) test and (5) Chi-square test. In this model, branches in the tree were divided *a priori* into foreground and background categories, and a likelihood ratio test was constructed to compare an alternative model that allows for some sites under positive selection on the foreground branches with a null model that does not. Tree topology was analyzed based on the concatenate and coalescent phylogenetic results, and the final species tree was used for the next analysis. The branch with *U. rockii* and *U. henryi* was selected as the foreground branch, and other branches were as a background branch. The rate (ω) of the non-synonymous substitution rate (dN) to synonymous substitution rate (dS) was used to measure the selective pressure. ω > 1 implies positive selection, ω = 1 means neutral selection and ω < 1 indicates negative selection (Yang and Nielsen, 2002). A likelihood ratio test (LRT) was constructed to compare the alternative model that allows sites to vary on the foreground branch with a null model that confined codon sites under neutral selection. The BEB method (Yang et al., 2005) was implemented to estimate the posterior probabilities for codon sites, and sites with a posterior probability larger than 0.9 were regarded as positively selected sites. The *p*-values were calculated with a Chi-square test and adjusted by the FDR method, and genes with *p*-values less than 0.05 were considered as positively selected genes (PSG). The Jalview software (Clamp et al., 2004) was used to view the amino acid sequences of some PSGs by highlighting the positively selected sites. The SWISS-MODEL (Schwede et al., 2003) was used to predict the three-dimensional protein structures and were further visualized with PyMOL software^5^. Furthermore, in order to detect more positive selected genes that involved in karst environment adaptation, a adaptive evolution was estimated by pairwise calculation of *K*_*a*_/*K*_*s*_ between all members of each clusters paralogous genes. Two paris of models: M1 (neutral) vs. M2 (selection) and M7 (beta) vs. M8 (beta + ω) were used in PAML package. For each pair of nested model the log likelihood values are compared using the likelihood ratio test (LRT).

Gene ontology (GO) enrichment analyses were conducted with detected *Urophysa* PSGs and adapted paralogous genes to infer their functional information using the DAVID function toll (Huang D. W. et al., 2008). The KOBAS software (Xie et al., 2011) was used to test the statistical enrichment of PSGs in the Kyoto Encyclopedia of Genes and Genomes (KEGG) pathways (Ogata et al., 2000). Moreover, the Cluster of Orthologous Groups of proteins (COG) database (Tatusov et al., 2000) was also used to predict the function of these PSGs. We then used a BLASTX search of these PSGs and Mul_genes with *Arabidopsis thaliana* to explore their homologies and functions.

## Results

### Morphological Characteristics and Growth Habit of *Urophysa* Species

*U. rockii* and *U. henryi* inhabit the karst cliffs and bloom in the winter, with the flourishing florescence between December to March. These species’ leaves possess obvious cuticular wax and the leaves color changes with the stages of development, which is green in the early stage of growth and purple or red in late development. Although these two species are herbaceous, they have long and stout rhizomes and are deeply rooted in the rock cracks. During field observations, we found that the pedicels of these two species bend toward the rock cracks in the fruiting period, and disperse seeds into the cracks of the karst rock. In contrast, the non-karst species *S. adoxoides*, *A. ecalcarata*, and *A. coerulea* grow in forest or roadside, although the three species also have rhizome (Tamura, 1995; Li, 2006). Leaves of the three species have not cuticular wax, and flowering times are from March to June and their seeds are mainly dispersed by gravity.

### Assembly Statistics and Function Annotation

In this study, a total of 46.19 million clean reads were generated, and a total of 702,823 contigs were collected in the non-redundant assembled transcriptomes (Table 1). There were 75,477 and 85,640 unigenes detected in *U. rockii* and *U. henryi* and the N50 value was 1,715 and 1,652 bp, respectively. The number of unigenes found in *A. ecalcarata* and *S. adoxoides* were 69,978 and 100,099 with the length of N50 ranging from 1,655 to 1,582 bp. We detected more unigenes and longer N50 length compared to other species in Ranunculaceae, such as *Helleborus thibetanus*, *Ranunculus* spp. and *Coptis chinensis* (Chen et al., 2015, 2017; Shi et al., 2019). The assembly completeness evaluation results showed that all assembled transcriptomes had high BUSCO completeness, which varied from 86.73 to 91.59% in the four species (Supplementary Figure 1). The clean data were submitted to the NCBI Sequence Reads Archive (SRA) database (accession number: SRR6816169, SRR6816171-SRR6816172, SRR6816174, SRR12805323, and SRR12805326). By annotation, there were 37,200 (49.29%, *U. rockii*) and 40,493 (47.28%, *U. henryi*) unigenes matches on the Flowering plant database, while there were 37,121 (53.05%) and 58,408 (58.35%) in *A. ecalcarata* and *S. adoxoides*, respectively. Additionally, there were 41.46–47.41% unigene matches on Swiss-Prot database and ranged from 26.69 to 28.03% on the Pfam database, please refer to Table 2 for detailed annotation information for the four species.

**TABLE 1:** Overview of the *de novo* assembly of the transcriptome of all species.

Species	*U. rockii*	*U. henryi*	*A. ecalcarata*	*S. adoxoides*
Number of clean reads	120,601,420	123,219,518	104,770,852	113,342,884
GC content (%)	43.6	43.3	44.38	41.5
Contig number	167,949	180,413	152,577	201,884
Maximum contig Length (bp)	15,723	15,788	16,889	13,819
Total unigenes generated	75,477	85,640	69,978	100,099
N50 (bp)	1,715	1,652	1,655	1,582
Total number of transcripts	167,949	180,413	152,577	201,884

**TABLE 2:** Statistics of annotations for assembled.

Terms	*U. rockii* number (%)	*U. henryi* number (%)	*A. ecalcarata* number (%)	*S. adoxoides* number (%)
Flowering plant	37,200 (49.29)	40,493 (47.28)	37,121 (53.05)	58,408 (58.35)
COG	23,452 (31.07)	28,685 (33.49)	22,128 (31.62)	33,641 (33.61)
Swiss-Prot	31,293 (41.46)	38,147 (44.54)	29,387 (41.99)	47,457 (47.41)
Pfam	20,149 (26.69)	22,906 (26.75)	19,160 (27.38)	28,057 (28.03)
GO	27,995 (37.09)	33,852 (39.53)	26,838 (38.35)	42,423 (42.38)
KEGG	24,570 (32.55)	30,015 (35.05)	23,047 (32.93)	37,606 (37.57)

### Identification of Orthologs, Paralogs, and Phylogenetic Analysis

**FIGURE 3:**
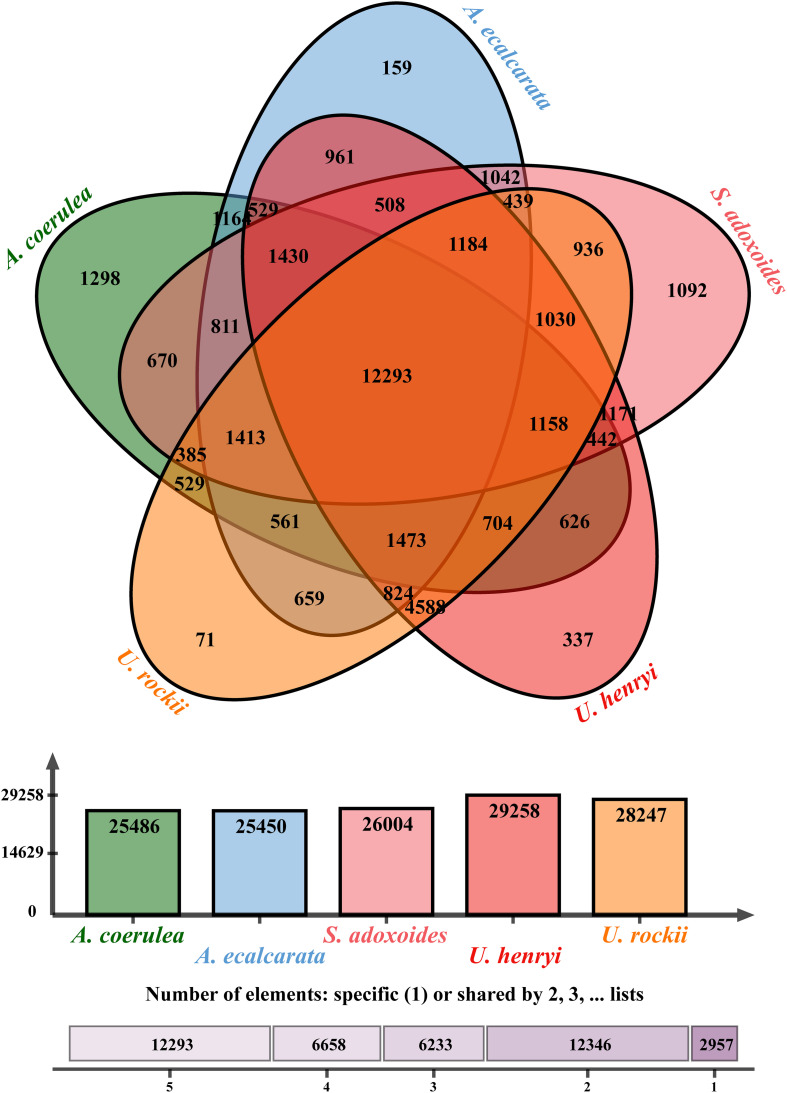
Venn diagram showing the numbers of orthologs identified in genus *Urophysa* and close species using OrthoVenn2 (https://orthovenn2.bioinfotoolkits.net/home).

**FIGURE 4:**
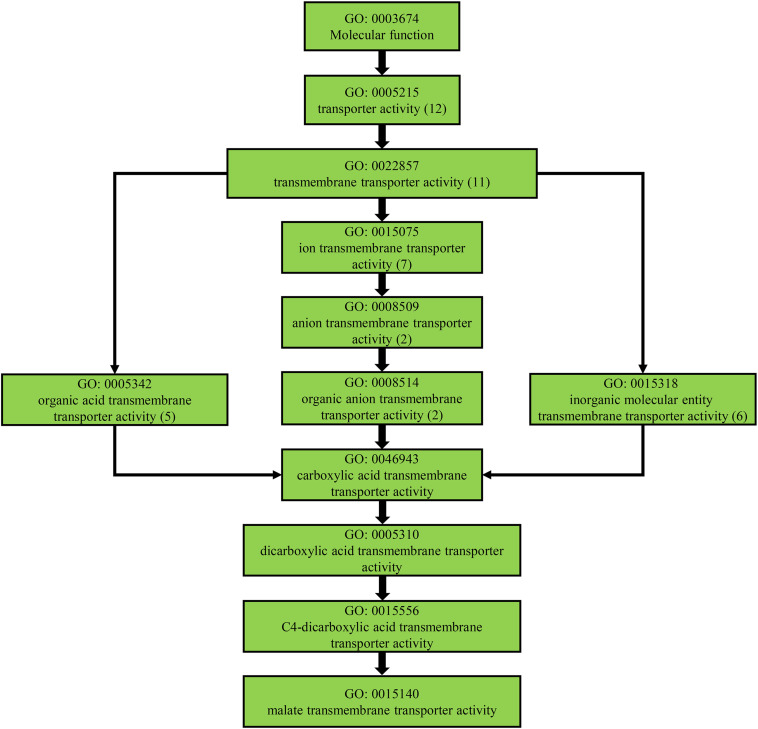
Transmembrane transporter activity related pathway detected from the positively selected genes of *Urophysa*.

**FIGURE 5:**
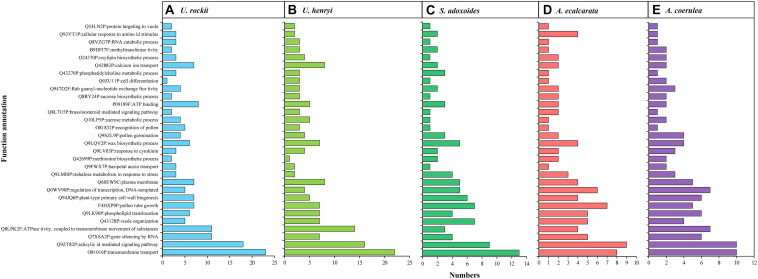
Function annotation for the 30 clusters of paralogous genes under significant adaptive evolution. **(A–E)** indicate the detail information of *U. rockii*, *U. henryi*, *S. adoxoides*, *A. ecalcarata* and *A. coerulea.*

From the orthologous analysis, a total of 40,487 putative orthologs were identified from the four species and *A. coerulea* based on OrthoMCL. There were 12,293 orthologs shared by all five species, and 4,588 orthologs were only found in the two *Urophysa* species (Figure 3). According to GO enrichment analysis, many orthologs shared by *U. rockii* and *U. henryi* were involved in response to stimuli, transport, ion binding, transporter activity, translation, membranes and others (Supplementary Figure 2). Among them, several metabolic processes were significantly enriched, for example, translation, RNA modification, proteolysis, DNA recombination, cytoplasmic translation, regulation of calcium-mediated signaling, nutrient reservoir (Supplementary Figure 3), and especially for the regulation of transmembrane transporter activity and calcium-mediated signaling (Figure 4 and Supplementary Figure 4). Meanwhile, 811 orthologs were exclusively found in the three non-karst species. All orthologs were non-significantly enriched and most of them were involved in metabolic process, molecular process, and macromolecule metabolic process (Supplementary Figure 5). In addition, 243 clusters of paralogous genes were detected among the five species, and by function annotation, many paralogs were related to transmembrane transport, salicylic id mediated signaling pathway, gene silencing by RNA, ATPase tivity (Figure 5 and Supplementary Table 1).

**FIGURE 6:**
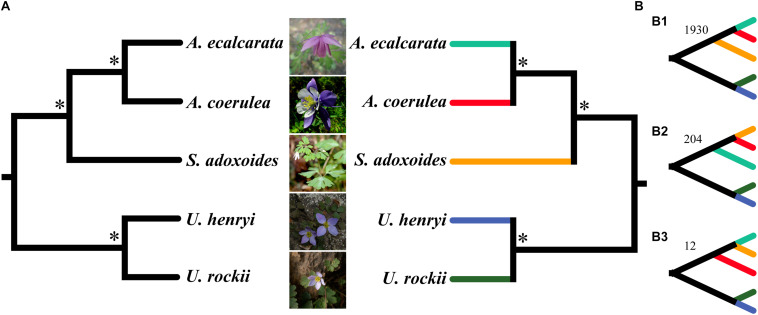
The phylogenetic analysis using concatenate **(A)** and coalescent method **(B)** based on single-copy genes. Asterisk (^∗^) above the branches in left **(A)** concatenate tree indicate the maximum support values and posterior probabilities in ML and BI analyses. ^∗^Above branches in right **(B)** coalescent tree are support values of MP-EST and STAR with bootstrapping analyses and local posterior probabilities. Panels **(B1–B3)** are the coalescent results with the 3 tree topologies and correspondent SCG numbers.

A total of 2,271 SCGs were filtered from the five species and used for phylogenetic analysis. Alignments of concatenated SCGs were 2,229,258 bp in length, including 126,095bp (5.65%) variable sites and 31,439bp (1.41%) parsimony-informative sites. ML and BI phylogenetic analyses showed that *S. adoxoides* was firstly differentiated when rooted *Urophysa* species as outgroup, and *A. ecalcarata* tightly cluster with *A. coerulea* (Figure 6A). The coalescent analysis detected 2,271 gene trees, and the final species tree was consistent with the result from concatenate method (Figure 6B). Phylogenetic signal exploration of each gene tree topology found that 1,930 gene trees (84.95%) shared the same topology (Figure 6B1), which was consistent with the topology of species tree (Figures 6A,B). Gene trees conflicts were also detected (Figure 6B2,B3), and the final species tree was used in next positive selection analysis.

### Positive Selection Analysis and Functional Annotation

**FIGURE 7:**
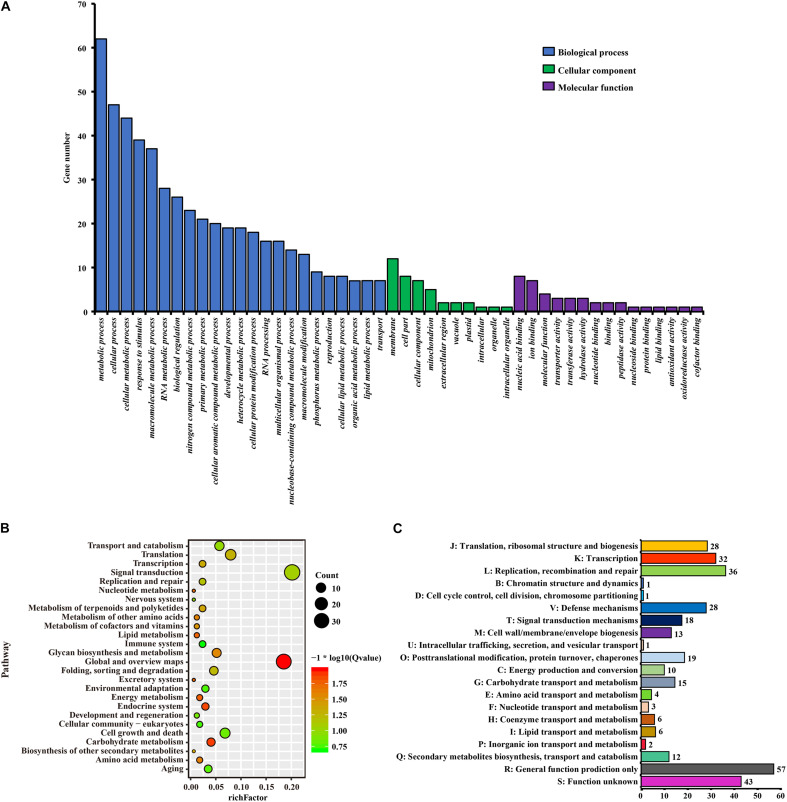
The function enrichment analyses of PSGs in *Urophysa* species. **(A)** GO annotation results; **(B)** KEGG annotation results; **(C)** COG annotation results.

A total of 335 SCGs exhibited significant positive selection (PSGs) (*P*-value < 0.05). We found that most of the PSGs were enriched in (GO terms) biological processes including metabolic process, stimulus-response, nitrogen compound metabolic process, membrane, nucleic acid/ion binding, transporter/transferase/hydrolase activity, ion/acid, and types of biological compound transmembrane transport (Figure 7A and Supplementary Table 2). KEGG function analysis indicated that most PSGs participated in pathways that related to signal transcription, transport and catabolism, translation, cell growth and death, carbohydrate metabolism, environmental adaptation, and aging (Figure 7B and Supplementary Table 3). The results of COG enrichment analysis indicated that many PSGs were involved in replication, recombination and repair, transcription, defense mechanisms, translation, ribosomal structure and biogenesis, signal transduction mechanisms, carbohydrate transport and metabolism, secondary metabolites biosynthesis, transport and catabolism, energy production and conversion (Figure 7C). Besides, through adaptive analysis for the 243 clusters of paralogous genes, we found 30 paralogous clusters were obvious enriched, and 3 clusters with *p* < 0.001, 5 clusters with *p* < 0.01 and 22 clusters with *p* < 0.05 in both M1–M2 and M7–M8 test, which were involved in transmembrane transport, salicylic id mediated signaling pathway, gene silencing by RNA, ATPase activity, wax biosynthetic process, ATP binding (Figure 5 and Supplementary Table 1).

**FIGURE 8:**
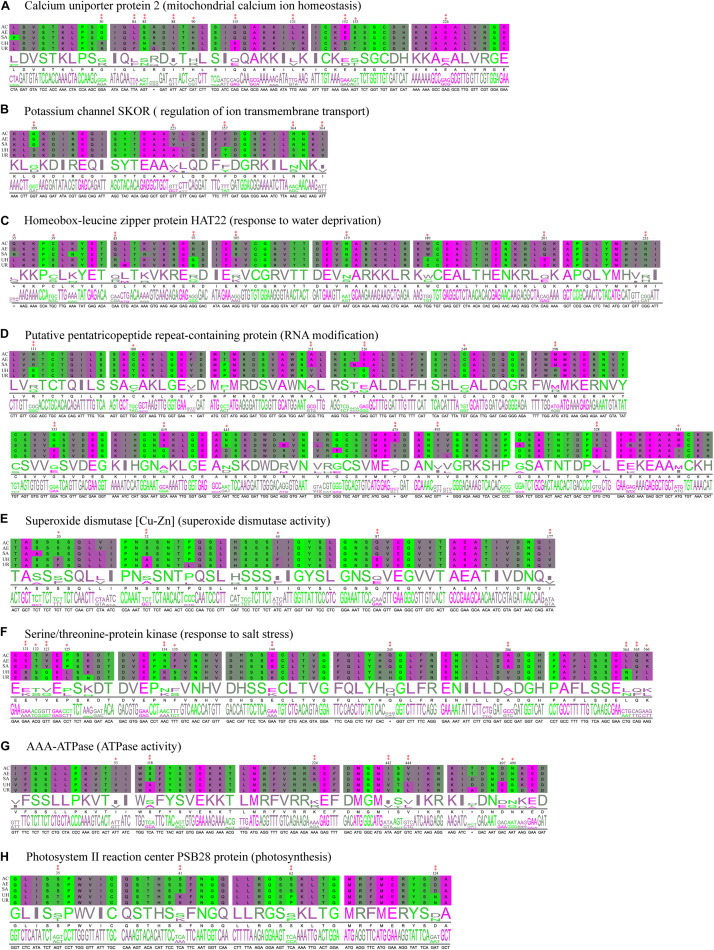
Partial alignment of some positively selected genes. **(A–C)** Positively selected genes (PSGs) related to transmembrane transport, ion homeostasis and water retention. **(D)** PSGs related to genetic information process. **(E,F)** PSGs related to the stress response. **(G,H)** PSGs related to photosynthesis and energy metabolism. Double red asterisks in the top stand for the amino acids is a positively selected site with a BEB posterior probability of more than 0.95, and one asterisk stands for the sites with a posterior probability larger than 0.90, but lower than 0.95. Numbers at the base of the asterisks stand for the site position in the PSGs. (AC, *A. coerulea*; AE, *A. ecalcarata*; SA, *S. adoxoides*; UH, *U. henryi* and UR, *U. rockii*).

**FIGURE 9:**
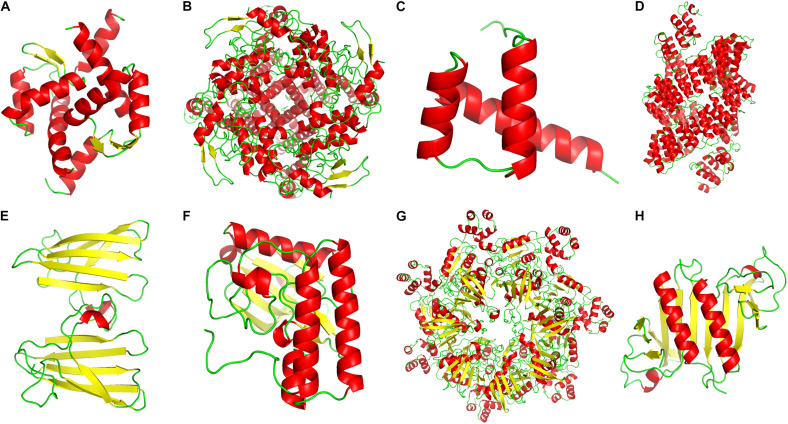
The secondary protein structures of some positively selected genes. **(A–C)** Positively selected genes (PSGs) related to transmembrane transport, ion homeostasis and water retention. **(D)** PSGs related to genetic information process. **(E,F)** PSGs related to the stress response. **(G,H)** PSGs related to photosynthesis and energy metabolism. Red color represented the α-helix, the yellow color stand for the β-sheet, and the green color stands for the β-turn.

By further functional annotation of positively selected genes and BLASTX with *A. thaliana*, many genes were related to the transmembrane transporter activity of ion/anion/organic anion/carboxylic acid and some were enriched with transmembrane transport (Figure 7 and Table 3), such as *At3g20240* (PSG_140) and *SKOR* (PSG_282). Other genes, included *At2g23790* (PSG_16), *CAMRLK* (PSG_168), are involved in the homeostasis of calcium ions were detected. We also found many genes related to water retention or water deprivation response and contributed to the osmotic stress response [such as *HAT22* (PSG_130), *CPK13* (PSG_95), *DRIP2* (PSG_162) and *LEA6* (PSG_305)]. Many of PSGs were found related to genetic information process [e.g., *PCMP-H14* (PSG_13), *FAS2* (PSG_175), *EXO1* (PSG_275)], stress response [*ATPK2* (PSG_198), *OXI1* (PSG_274), *At5g58480* (PSG_323)], photosynthesis and energy metabolism [*ndhS* (PSG_143), *GLYK* (PSG_154), *PSB28* (PSG_183)]. Detailed annotation information is listed in Supplementary Table 2. Several PSGs with their positively selected sites and three-dimensional protein structures were visualized and are shown in Figures 8, 9. The illustrated PSGs were involved in transmembrane transport, ion homeostasis and water retention (Figures 8A,B, 9A,B), genetic information process (Figures 8D, 9D), stress response (Figures 8E,F, 9E,F), photosynthesis and energy metabolism (Figures 8G,H, 9G,H). Most of the positively selected sites that were detected had BEB posterior probabilities of more than 0.90. The three-dimensional protein structures of PGSs mainly constituted an α-helix and β-sheet. Additionally, some paralogs were also involved in transmembrane transport, calcium ion transport (e.g., *ABC* family genes), and energy metabolism (e.g., *HSP70*) (Table 3).

**TABLE 3:** Positively selected genes (PSGs) and clusters of paralogous genes (Mul_gene) related to ion homeostasis and water retention, genetic information process, stress response, photosynthesis, and energy metabolism in *Urophysa* species.

PSG Name	Swiss Prot ID	Gene	Product	Function	Adjust *P*-value
**Transmembrane transport, ion homeostasis and water retention**					
PSG_16	O64823	*At2g23790*	Calcium uniporter protein 2, mitochondrial	Mitochondrial calcium ion homeostasis	9.95E-03
PSG_130	P46604	*HAT22*	Homeobox-leucine zipper protein HAT22	Response to water deprivation	4.04E-02
PSG_140	Q9LJX5	*At3g20240*	Probable mitochondrial adenine nucleotide transporter BTL1	Transmembrane transporter activity	4.49E-02
PSG_165	Q9XYL0	*fcpA*	Probable C-terminal domain small phosphatase	Hyperosmotic response	2.07E-02
PSG_168	Q9FK63	*CAMRLK*	Calmodulin-binding receptor kinase CaMRLK	Response to osmotic stress	2.38E-02
PSG_282	Q9M8S6	*SKOR*	Potassium channel SKOR [Stelar K (+) outward rectifying channel]	Regulation of ion transmembrane transport	3.04E-02
PSG_95	Q9FXQ3	*CPK13*	Calcium-dependent protein kinase 13	Response to water deprivation	1.03E-02
PSG_162	Q94AY3	*DRIP2*	E3 ubiquitin protein ligase DRIP2	Response to water deprivation	1.14E-03
PSG_305	Q39138	*LEA6*	Late embryogenesis abundant protein 6	Rsponse to osmotic stress	3.93E-03
Mul_46	O81016	*ABCC10*	ABC transporter C family member 10	Transmembrane transport	1.57E-04
Mul_132	Q8LPK2	*ABCB2*	ABC transporter B family member 2	ATPase tivity, coupled to transmembrane movement of substances	1.14E-03
Mul_178	Q9FWX7	*ABCB11*	ABC transporter B family member 11	Basipetal auxin transport	1.38E-02
Mul_92	Q42883	*LCA1*	Calcium-transporting ATPase, endoplasmic reticulum-type	Calcium ion transport	3.95E-02
Mul_93	Q43128	*AHA10*	ATPase 10, plasma membrane-type	Vuole organization	2.05E-03
**Genetic information process**					
PSG_13	Q9FND7	*PCMP-H14*	Putative pentatricopeptide repeat-containing protein	RNA modification	2.07E-02
PSG_175	Q6ZD63	*FAS2*	Chromatin assembly factor 1 subunit FAS2 homolog	DNA repair	1.29E-03
PSG_213	Q9FZA4	*DOF1.4*	Dof zinc finger protein DOF1.4	regulation of transcription, DNA-templated	2.99E-02
PSG_275	Q8L6Z7	*EXO1*	Exonuclease 1	DNA repair	3.30E-02
PSG_8	F4JNY0	*APE2*	DNA-(apurinic or apyrimidinic site) lyase 2	DNA repair	2.08E-02
**Stress response**					
PSG_198	Q39030	*ATPK2*	Serine/threonine-protein kinase AtPK2/AtPK19	Response to salt stress	3.16E-02
PSG_274	Q9LSF1	*OXI1*	Serine/threonine-protein kinase OXI1	Response to wounding	2.68E-02
PSG_288	Q40374	*PR-1*	Pathogenesis-related protein PR-1	Response to biotic stimulus	3.41E-02
PSG_323	Q9FGH4	*At5g58480*	Glucan endo-1,3-beta-glucosidase 9	Defense response	4.32E-02
PSG_146	O04997	*SODCP*	Superoxide dismutase [Cu-Zn], chloroplastic	Superoxide dismutase activity	2.50E-04
**Photosynthesis and energy metabolism**					
PSG_143	Q9T0A4	*ndhS*	NAD(P)H-quinone oxidoreductase subunit S, chloroplastic	Photosynthetic electron transport chain	4.29E-02
PSG_154	Q944I4	*GLYK*	D-glycerate 3-kinase, chloroplastic	Aphotorespiration	1.54E-02
PSG_158	Q9SNQ6	*HD3B*	Protein HEADING DATE 3B	Positive regulation of short-day photoperiodism, flowering	6.05E-03
PSG_181	Q9LH84	*At3g28510*	AAA-ATPase At3g28510	ATPase activity	3.62E-04
PSG_183	Q0JG75	*PSB28*	Photosystem II reaction center PSB28 protein, chloroplastic	Photosynthesis	2.68E-02
Mul_52	P09189	*HSP70*	Heat shock cognate 70 kDa protein	ATP binding	2.26E-02

## Discussion

Adaptation is an evolutionary process that allows organisms to survive in their habitat better through natural selection (Lan et al., 2017). Recently, transcriptome data of many non-model species have been obtained and employed for studies of species polyploid, species divergence and differential gene expression (Wang H.F. et al., 2019; Hina et al., 2020; Li et al., 2020), phylogenetic and phylogenomic analysis (Pouchon et al., 2018; Opatova et al., 2019) as well as for detecting natural selection and exploring adaptive evolution in closely related species (Ma et al., 2019; Zeng et al., 2019; Peng et al., 2020; Xie et al., 2020). To date, however, most transcriptome studies have been undertaken on model species and studies of adaptation to the karst environment are not well understood. This is the first study to explore the adaptation mechanisms of species in the karst floristic region through transcriptome data, which will offer excellent opportunities to explore the processes of adaptive evolution.

### Stresses or Stimuli Resistance

Stresses and stimuli, such as drought stress, salinity stress, extreme temperatures, chemical toxicity and oxidative stress are serious threats to species and result in the deterioration of the environment (Wang and Altman, 2003). Karst limestones are sedimentary rock outcrops that consist primarily of calcium carbonate and are generally characterized by poor soil development, low soil water content, periodic water deficiency and heat stress (Kang et al., 2014). Under such hostile conditions, plant uptake of nutrients (such as nitrogen and phosphorus) may be restricted, which greatly threaten plant growth, development and survival (Wang et al., 2014). We found that through the functional annotation of shared orthologs (4,588) (Figure 3) and PSGs in two karst species (*U. rockii* and *U. henryi*) (Figure 7), genes associated with stress resistance, DNA repair, ion transmembrane transport and homeostasis maintenance were overrepresented (Table 3 and Supplementary Tables 1–3). Among them, a large proportion of PSGs were involved in various stresses or stimulus resistance, such as salt stress, oxidative stress, cold and wounding.

Abiotic stresses are often interconnected and may induce similar cellular damage (Wang and Altman, 2003). For example, oxidative stress, which frequently accompanies high temperature, salinity, or drought stress, may cause denaturation of functional and structural proteins (Smirnoff, 1998). Through a BLASTX search of these PSGs in *A. thaliana*, PSG_146 was homologous to *SODCP* (Table 3), which plays an important role in superoxide dismutase activity (Abarca et al., 2001) and it can destroy radicals that are normally produced within the cells and toxic to biological systems (Sunkar et al., 2006). We found that some PSGs were involved in DNA repair, recombination, and RNA modification, which are essential for maintaining genomic stability in organisms (Zhang L. et al., 2013). For example, PSG_175 and PSG_275 were, respectively, homologous to *FAS2* and *EXO1*, which are involved in the repair of DNA double-strand breaks (DBSs) via homologous recombination (Mozgová et al., 2010) and DNA mismatch repair (Schaetzlein et al., 2007), respectively. These PSGs may allow the *Urophysa* species to overcome the stresses and stimuli in the karst environment.

### Homeostasis Related Genes in Karst Adaptation

Because of the relatively higher concentrations of calcium (Ca^2+^) and magnesium (Mg^2+^), higher pH levels and a lower water storage capacity in karst limestone bedrock compared with non-karst soils (Nie et al., 2011; Hao et al., 2015), *U. rockii* and *U. henryi* must confront highly alkaline conditions, thin soil layers, and desiccation on porous limestone bedrock. To address this issue, the two species must regulate ion and water transport to maintain osmotic balance. We found more orthologs in the two karst species (4,588) than other three non-karst species (811) (Figure 3), Additionally, our GO enrichment analysis found more orthologs involved in calcium-mediated signaling, nitrogen/phosphorus compound metabolic process, membrane, transport, ion binding, and nutrient reservoir activity (Figure 4 and Supplementary Figure 3). Many PSGs and Mul_genes that were related to the membrane, transmemberane transport, calcium ion transport, transport and ion homeostasis were detected (Figures 5, 7, Table 3, and Supplementary Figure 4). The three-dimensional protein structures of these PGSs were mainly composed of an α-helix and β-sheet, and numerous positively selected sites existed in their amino acid sequences, which might suggest that these genes undergo strong natural selection and contribute to the karst environment adaptation of *Urophysa* species.

The homeostasis of intracellular ion concentrations is fundamental to the physiology of living cells. Proper regulation of ion flux is necessary for cells to keep the concentrations of toxic ions low and to accumulate essential ions (Zhu, 2001). Positively selected gene *SKOR* (Table 3), a highly selective outward-rectifying potassium channel, plays an important role in the regulation of ion transmembrane transport (Frédéric et al., 1998). Previous studies have revealed that the outwardly rectifying K^+^ channel, which is coded by the *SKOR* gene, involves in K^+^ release into the xylem sap toward the shoots, a critical step in the long-distance distribution of K^+^ from roots to the upper parts of the plant (Johansson et al., 2006; Liu et al., 2006). Thus, the potassium channel coded by the *SKOR* gene may assist in regulating homeostasis in *U. rockii* and *U. henryi* in karst habitat. Notably, we found genes PSG_16 and PSG168 that coded the calcium uniporter protein 2 and calmodulin-binding receptor kinase *CaMRLK*, respectively. Furthermore, we also found some paralogs (e.g., Mul_92 is homologous to gene *LCA1*) that were associated with calcium ion transport from the cytosol to an endomembrane compartment (Wimmers et al., 1992) (Table 3). The calcium uniporter mediates calcium uptake into mitochondria and regulates the mitochondrial calcium ion homeostasis, which thereby plays a key roles in cellular physiology and regulates cell bioenergetics, cytoplasmic calcium signals and activation of cell death pathways (De Stefani et al., 2011). The Calmodulin-binding receptor kinase *CaMRLK* is involved in auxin and osmotic stress response (Charpenteau et al., 2004). Moreover, PSGs involved in water deprivation response and hyperosmotic tolerance were detected in this study, such as *HAT22*, *CPK13*, *DRIP2*, *LEA6*, and *fcpA*. Among them, *CPK13* plays an important role in cellular water deprivation (Saijo et al., 2010), which might function in signal transduction pathways that positively regulate responses to cold, salt and drought stresses. *LEA6* is involved in the adaptive response of water deficit for vascular plants (Olvera-Carrillo et al., 2010), which may partially explain why the two *Urophysa* species were able to inhabit habitats with a high level of aridity. All the calcium-regulator and water-transporter-related genes may play key roles for *U. rockii* and *U. henryi* in adapting to the extremely hostile karst environment with severe water deprivation and violent osmosis. Moreover, some paralogs (e.g., Mul_46, Mul_132, and Mul_178) belong to *ABC* transporter family, and relate to transmembrane transport (Bessire et al., 2011). We also found many membrane related genes from functional annotation of orthologs shared by *Urophysa* species and PSGs (Supplementary Figure 2), which also suggests that membrane systems play an important role in osmotic response to the karst environment.

### Morphological Adaptation of *Urophysa* Species

Previous studies suggested that the genus *Urophysa* has a close relationship with *Semiaquilegia* and *Aquilegia* and shared a common ancestor (Wang, 1992). Among them, *Urophysa* species possess obvious cuticular wax on their leaves’ surfaces, which was absent in other three non-karst species. The cuticular wax plays a vitally important role in protecting plant tissue from environmental stresses, limiting non-stomatal water loss, and helping to prevent the germination of pathogenic microbes (Raven and Edwards, 2004; Samuels et al., 2008; Zhang et al., 2010). Furthermore, through scanning electron micrographs of *Urophysa* species’ leaf epidermis in our previous study (Xie et al., 2017), numerous epidermal hairs were detected. Previous studies suggested that the epidermal hairs can increase the resistance of plants to herbivorous insects (Reymond et al., 2004; Clauss et al., 2006), and others found that epidermal hairs play important roles in the defense and protection of plants by adjusting the content of defense-related compounds (Traw and Bergelson, 2003; Jakoby et al., 2008). Additionally, species of *Aquilegia*, *Semiaquilegia*, and *Urophysa* have rhizomes, however, compared to the non-karst species, *Urophysa* species possess longer and stout rhizomes and are deeply rooted into the rock cracks, which can help them absorb nutrients and water efficiently (Xie et al., 2017). Moreover, through field investigation, we found that these two species disperse seeds into the karst cracks by bending their pedicels toward the rock cracks in the fruiting period, which allows more seeds to enter the cracks. This reproductive approach might ensure *Urophysa* species better adapt to the karst habitat. Phenotypic plasticity of *Urophysa* species associated with karst environment adaptation was also found by Xie et al. (2017) who identified extensive footprints of local adaptation from *U. rockii* and *U. henryi* specialized morphologies, including unusual floral organs, such as petaloid sepals that can display various colors in different flower phases, petals with a nectar spur that could attract more pollinators, and a mass of small seeds (thousand seed weight is 0.6684 ± 0.0038 g) (Zhang et al., 2013b). All of these specialized morphologies are likely to contribute to enhance reproductive efficiency, but also be favorable to adaptating to the harsh karst environment.

## Conclusion

Our transcriptomic analysis detected many PSGs and clusters of paralogous genes in *Urophysa* species. Among them, genes related to stress and stimulus resistance, transmembrane transport, calcium ion transport, cellular ion homeostasis, calcium signaling regulation and water retention were detected, which allow better adaptation to the karst environment. Several phenotypic characteristics, such as obvious cuticular wax, numerous epidermal hairs, long and stout rhizomes and unusual flowers, may also contribute to habitat adaptation. Our results showed that *Urophysa* species may have evolved successful strategies for karst environment adaptation.

## Data Availability Statement

The datasets generated for this study can be found in online repositories. The names of the repository/repositories and accession number(s) can be found in the article/Supplementary Material.

## Author Contributions

D-FX, R-YC, and X-JH designed the study. D-FX and X-YZ collected the materials and planned the experimental setup. D-FX, R-YC, and XF conducted bioinformatic analyses and drafted the manuscript. D-FX, MP, Y-LL, and C-BW revised the manuscript. All authors contributed to the article and approved the submitted version.

## Conflict of Interest

The authors declare that the research was conducted in the absence of any commercial or financial relationships that could be construed as a potential conflict of interest.

## Footnotes

1 http://www.bioinformatics.babraham.ac.uk/projects/fastqc/

2 https://busco-data.ezlab.org/v4/data/lineages

3 https://github.com/TransDecoder/TransDecoder/releases

4 http://www.phytozome.net/

5 https://pymol.org/2/

## Supplementary Material

Supplementary Figure 1 The assembly completeness test for the transcriptome of each species.

Supplementary Figure 2 Gene Ontology (GO) annotation for shared orthologs from *Urophysa rockii* and *Urophysa henryi*.

Supplementary Figure 3 Gene Ontology function analysis of the significantly enriched orthologs shared from *U. rockii* and *U. henryi*.

Supplementary Figure 4 The directed acycling graph revealed from the shared orthologs from *U. rockii* and *U. henryi*.

Supplementary Figure 5 Gene Ontology annotation for shared orthologs from non-karst species *Semiaquilegia adoxoides*, *Aquilegia ecalcarata*, and *Aquilegia coerulea*.

Supplementary Table 1 The function annotation and adaptive significance test for the 243 clusters of paralogous genes.

Supplementary Table 2 The GO annotation for all 335 PSGs detected from *Urophysa* species.

Supplementary Table 3 The KEGG annotation results for 335 PSGs detected from *Urophysa* species.
