# Supplementary figures and images for: A Combined Morphological and Molecular Evolutionary Analysis of Karst-Environment Adaptation for the Genus Urophysa (Ranunculaceae)
Source: Front Plant Sci. 2021 Jun 10;12:667988. doi: 10.3389/fpls.2021.667988 (PMC8223000; doi:10.3389/fpls.2021.667988)

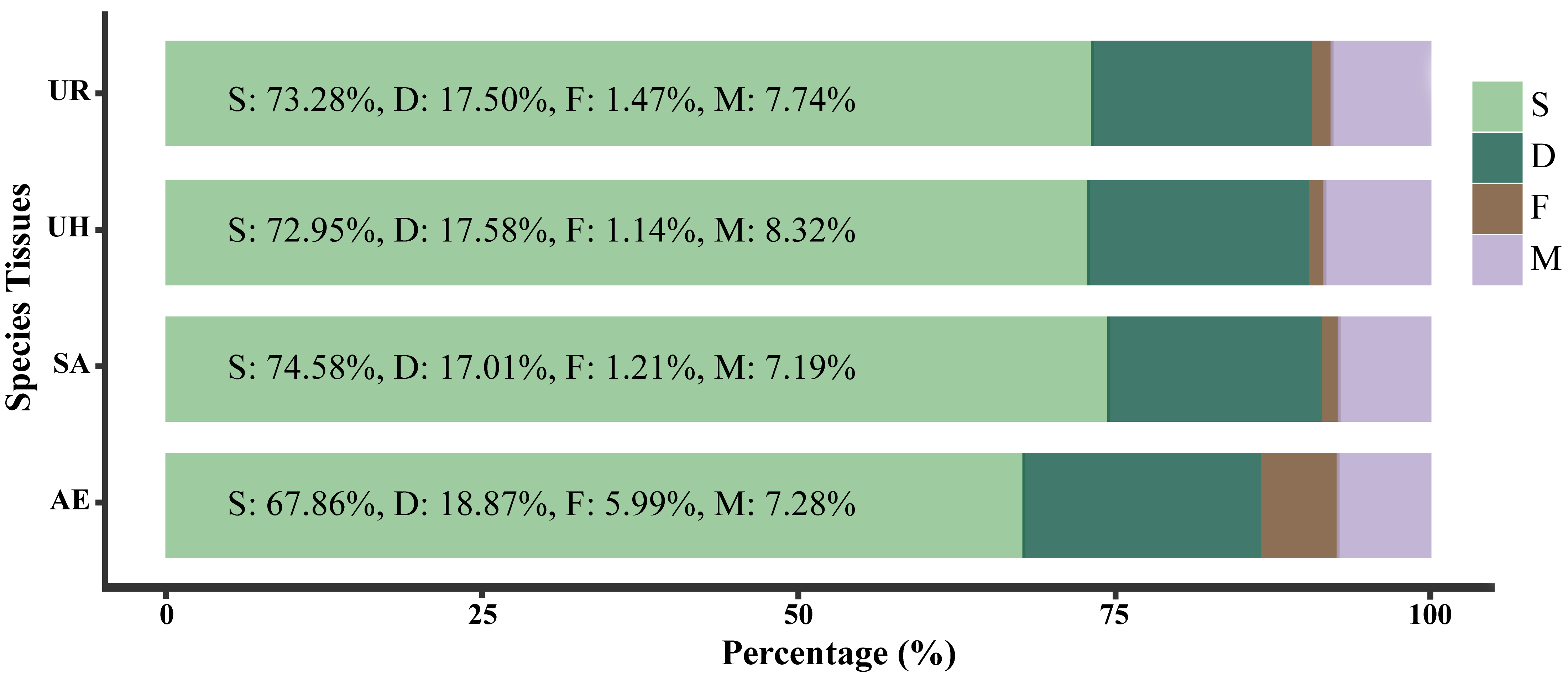

Supplement: Supplementary Figure 1 — The assembly completeness test for the transcriptome of each species. [file Image_1.TIF]

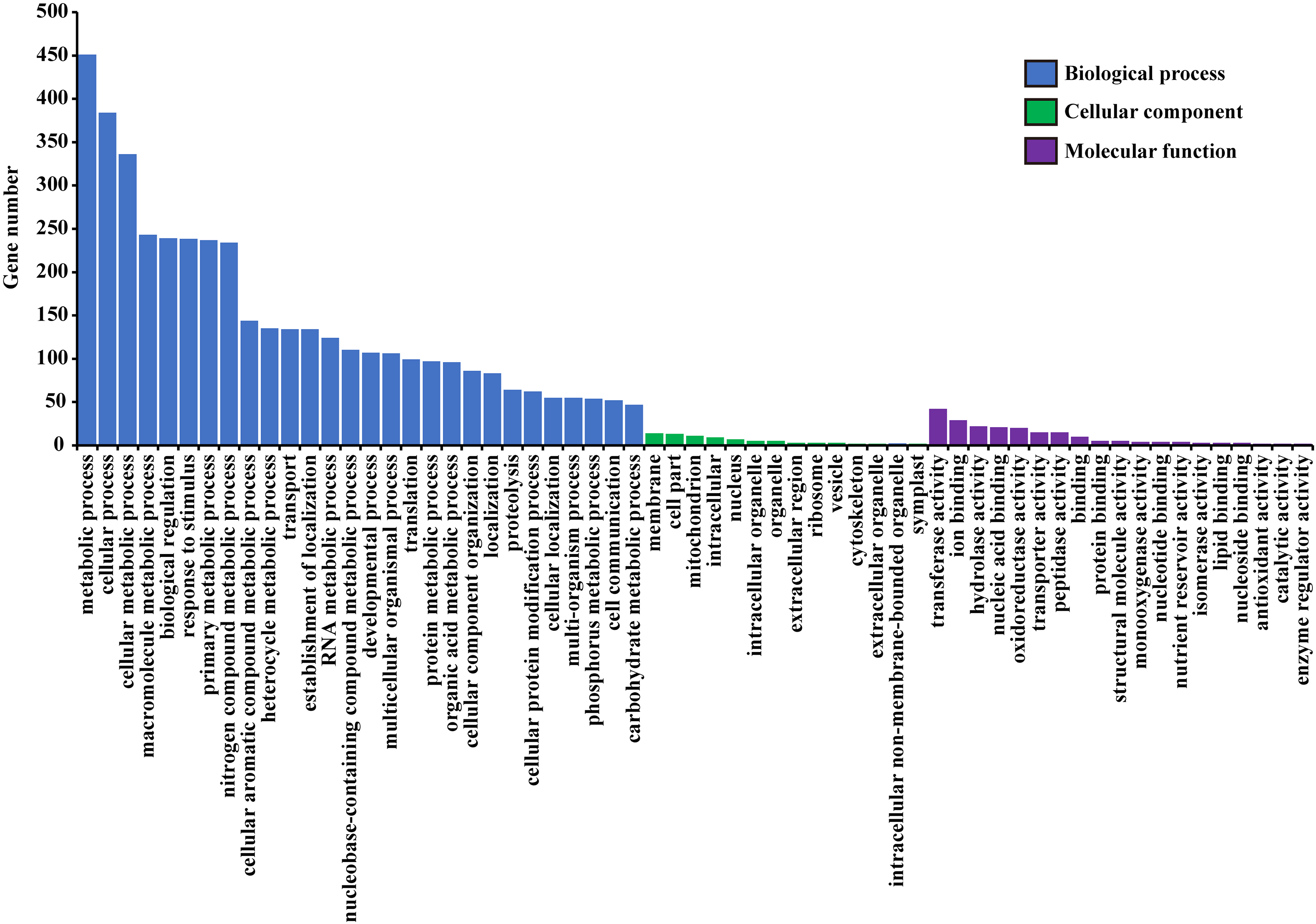

Supplement: Supplementary Figure 2 — Gene Ontology (GO) annotation for shared orthologs from Urophysa rockii and Urophysa henryi. [file Image_2.TIF]

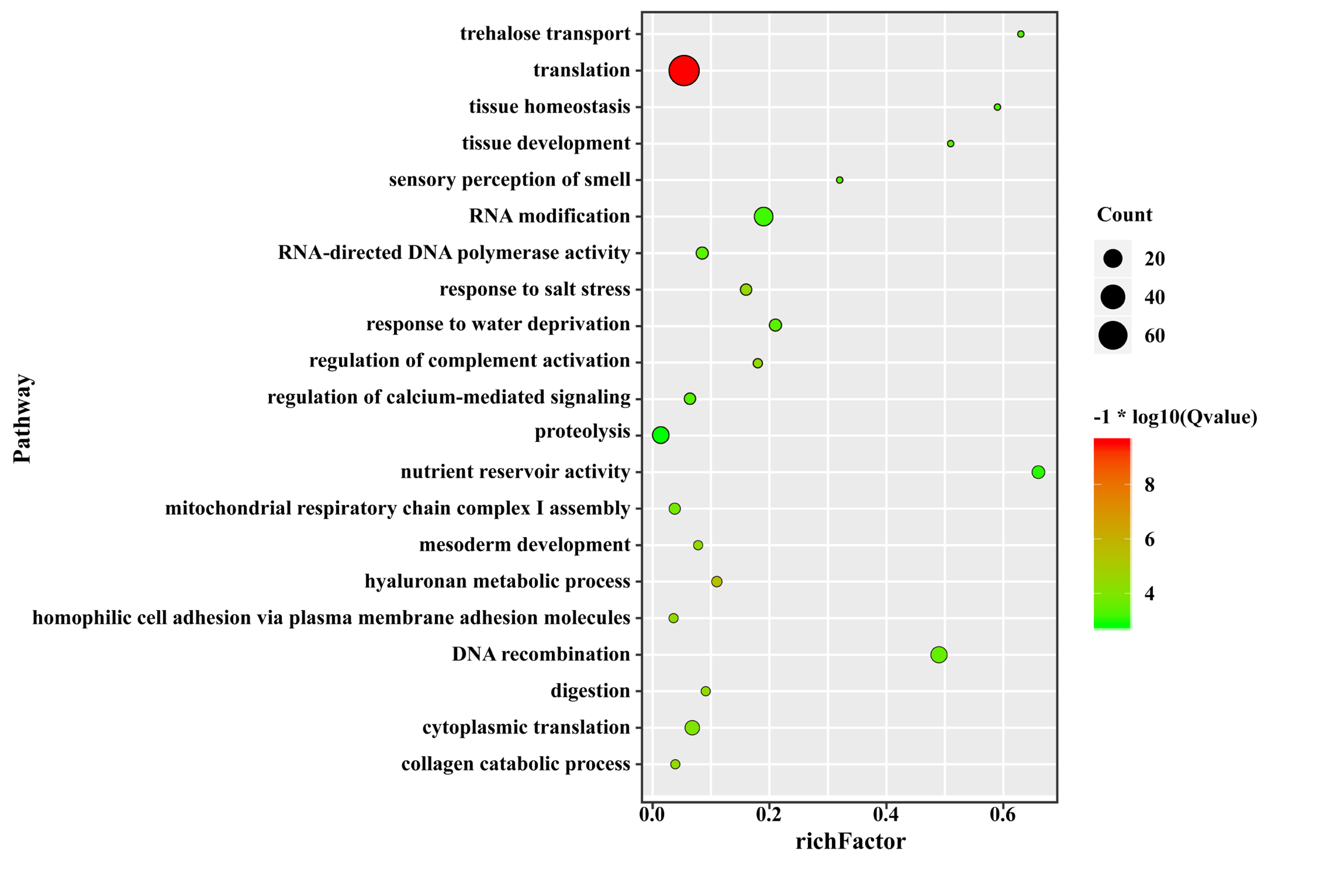

Supplement: Supplementary Figure 3 — Gene Ontology function analysis of the significantly enriched orthologs shared from U. rockii and U. henryi. [file Image_3.TIF]

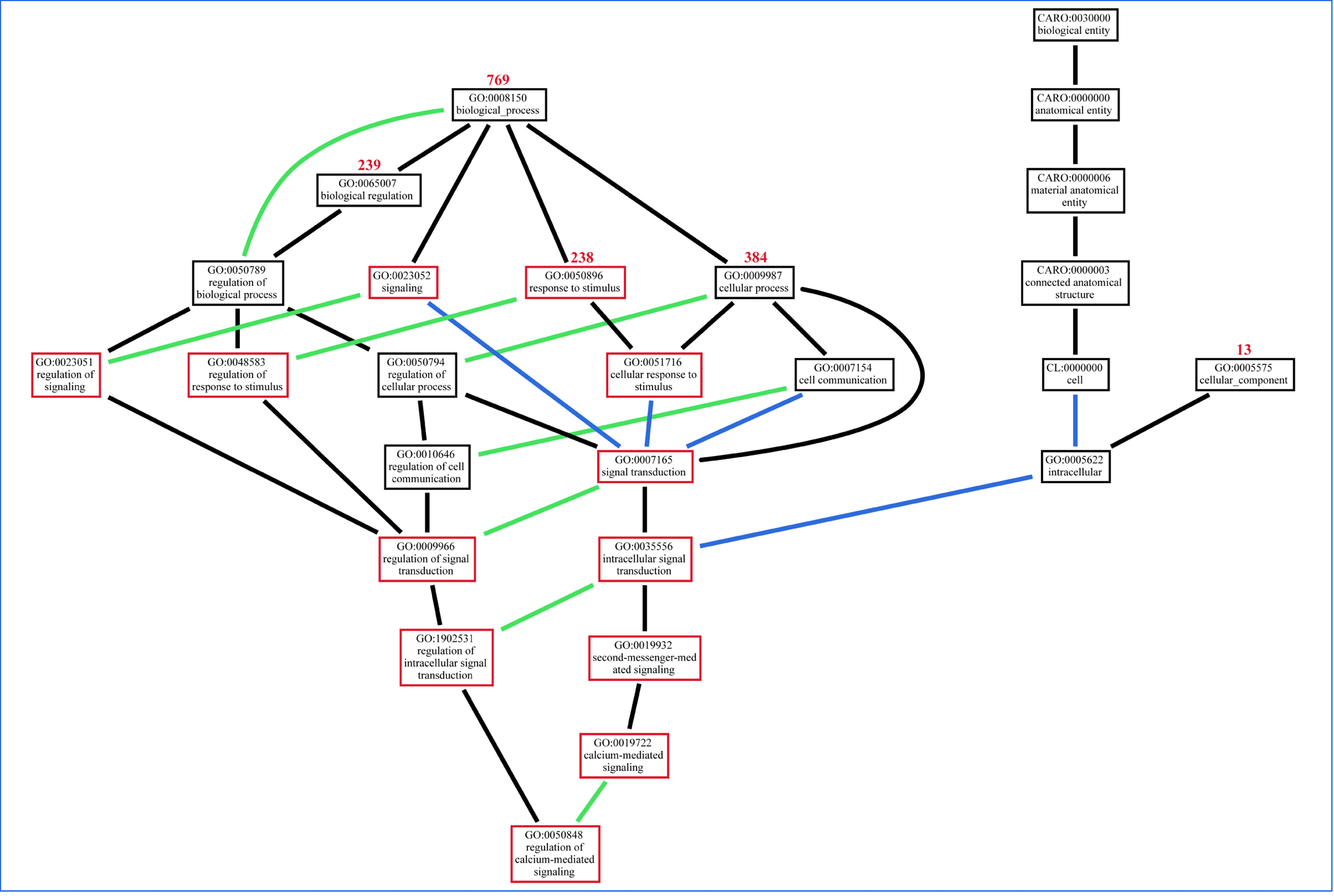

Supplement: Supplementary Figure 4 — The directed acycling graph revealed from the shared orthologs from U. rockii and U. henryi. [file Image_4.TIF]

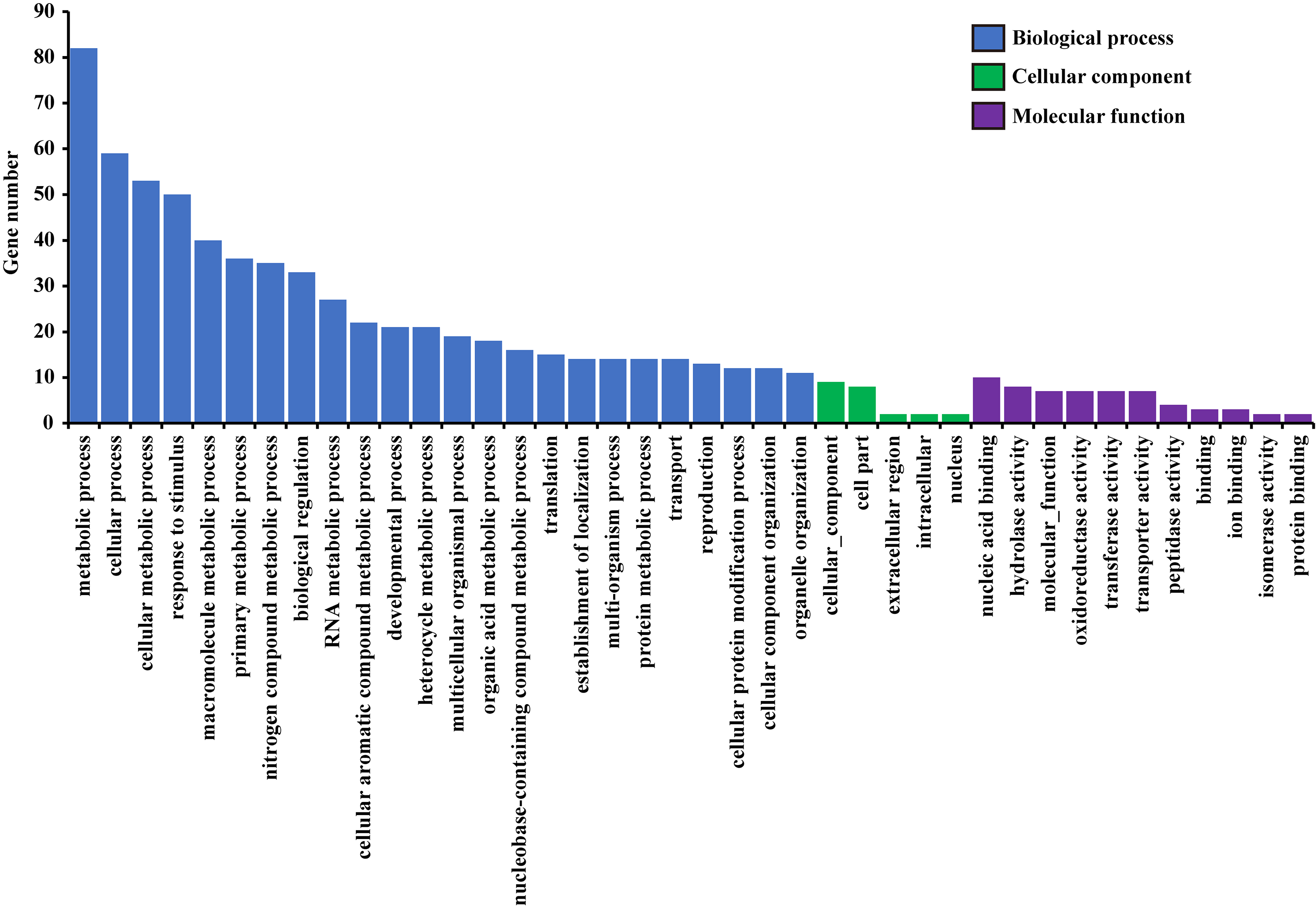

Supplement: Supplementary Figure 5 — Gene Ontology annotation for shared orthologs from non-karst species Semiaquilegia adoxoides, Aquilegia ecalcarata, and Aquilegia coerulea. [file Image_5.TIF]
